# Comparison of Prescribing Patterns Before and After Implementation of a National Policy to Reduce Inappropriate Alprazolam Prescribing in Australia

**DOI:** 10.1001/jamanetworkopen.2019.11590

**Published:** 2019-09-18

**Authors:** Andrea L. Schaffer, Nicholas A. Buckley, Rose Cairns, Sallie Pearson

**Affiliations:** 1 Centre for Big Data Research in Health, University of New South Wales Sydney, Sydney, Australia; 2 School of Medical Sciences, University of Sydney, Sydney, Australia; 3 School of Pharmacy, University of Sydney, Sydney, Australia; 4 New South Wales Poisons Information Centre, The Children’s Hospital at Westmead, Sydney, Australia; 5 Menzies Centre for Health Policy, University of Sydney, Sydney, Australia

## Abstract

**Importance:**

Benzodiazepines have been a common target for policy interventions to curtail inappropriate use, with mixed results. To reduce alprazolam misuse, in February 2017, Australia delisted the 2-mg tablet strength from public subsidy, eliminated refills, and reduced the pack size from 50 tablets to 10 tablets.

**Objective:**

To describe changes in alprazolam dispensing, prescribing, and poisonings associated with the implementation of a new policy to reduce inappropriate prescription of alprazolam in Australia.

**Design, Setting, and Participants:**

This interrupted time series analysis and cross-sectional study included data from a 10% sample of Australian people who received publicly subsidized dispensing claims and prescribing approvals for alprazolam from January 1, 2015, to December 31, 2018, and all calls to a poison information service involving alprazolam from February 1, 2015, to October 31, 2018. Autoregressive error models were used to quantify changes over time and compare patterns of use before and after the intervention. Data analyses were conducted from November 2018 to May 2019.

**Exposure:**

Implementation of the policy change on February 1, 2017.

**Main Outcomes and Measures:**

Monthly trends in alprazolam prescribing approvals and dispensings, quarterly trends in telephone calls involving alprazolam to a poison information service, and patterns of prescribing and dispensing before and after the intervention.

**Results:**

From 2015 to 2018, there were 71 481 alprazolam dispensings to 6772 people. After the intervention, overall dispensing decreased by 51.2% (95% CI, 50.5%-51.9%) and prescribing approvals increased by 17.5% (95% CI, 13.0%-22.2%). Overall, the proportion of dispensing of packs of 51 to 100 tablets increased from 5776 of 24 282 dispensings (23.8%) to 4888 of 10 676 dispensings (45.8%) (risk difference [RD], 22.0% [95% CI, 19.4%-24.6%]) and dispensing of packs of more than 100 tablets increased from 1029 of 24 282 dispensings (4.2%) to 1774 of 10 676 dispensings (16.6%) (RD, 12.4% [95% CI, 10.6%-14.2%]). Among people receiving their first alprazolam prescription, initiation with packs of 10 tablets or fewer increased from 16 of 1127 people (1.4%) before the intervention to 139 of 589 people (23.6%) after the intervention (RD, 22.2% [95% CI, 18.7%-25.7%]). Alprazolam treatment initiation with packs of more than 50 tablets also increased from 63 of 1127 people (5.6%) before the intervention to 144 of 589 people (24.4%) after the intervention (RD, 18.9% [95% CI, 15.1%-22.6%]). During 1 year before the intervention, patients received a median (interquartile range [IQR]) total of 250 (50-600) tablets and median (IQR) total combined tablet strength of 188 (50-550) mg. During 1 year after the intervention, people were dispensed less alprazolam, with a median (IQR) total of 200 (50-500) tablets and median (IQR) total combined tablet strength of 120 (30-360) mg. There was little change in poisoning calls involving alprazolam.

**Conclusions and Relevance:**

This study found that after the policy intervention, subsidized alprazolam use decreased, but the increase in prescribing approvals placed additional burden on prescribers. Even after the intervention, most people who were dispensed alprazolam were still receiving treatment contrary to best-practice recommendations. Furthermore, the poison information center data suggested that people were still being dispensed the 2-mg tablet strength, presumably as nonsubsidized (ie, private) prescriptions.

## Key Points

**Question:**

Was implementation of changes to public subsidy of alprazolam associated with changes in alprazolam prescribing, dispensing, or poisonings?

**Findings:**

In this interrupted time series analysis and cross-sectional study, after the February 2017 implementation of the policy intervention, subsidized dispensings decreased by 51.2% but prescribing approvals increased by 17.5%. Despite the policy reducing pack size, dispensing of more than 50 tablets also increased overall and among people receiving new alprazolam prescriptions.

**Meaning:**

This study found a reduction in subsidized alprazolam use after the policy change, but prescribing contrary to best-practice recommendations was common, with a likely shift to the private market.

## Teaser

This interrupted time series analysis and cross-sectional study examines changes in alprazolam prescribing patterns after a national policy change that reduced alprazolam subsidies in Australia.

## Introduction

Benzodiazepines have been a frequent target for policy interventions owing to long-standing evidence about overprescribing and misuse.^1,2,3,4^ A common approach used by third-party payers to reduce inappropriate use of prescribed medications is to restrict the conditions under which medications are reimbursed.^5,6,7^ While these policies have the potential to change the way in which medications are prescribed and used, they are relatively imprecise tools that can also lead to unintended consequences.^8,9,10^ Previous policy interventions targeting benzodiazepines have had mixed results, as they did not selectively target people who were inappropriately prescribing or misusing benzodiazepines. While policy interventions are often associated with a reduction in overall benzodiazepine use, these shifts in prescribing behavior often occur across the entire treated population and, in many cases, among people for whom use is considered appropriate.^11,12^ In recent years, there has been a renewed interest in benzodiazepine harms due to their use in combination with opioids, which greatly increases the risk of fatal overdose.^13,14,15^

Alprazolam, a short-acting benzodiazepine, is subsidized in Australia for treatment of panic disorder when other treatments have failed or are inappropriate. It is significantly more toxic than other benzodiazepines^16^ but with no demonstrable additional therapeutic benefit.^17^ It is also misused frequently by people who inject drugs^18,19^ and commonly present in drug overdose.^20,21,22^ Concerns have been raised about inappropriately large pack sizes and tablet strengths, and in February 2017, in an attempt to reduce the inappropriate use of alprazolam, the Australian Pharmaceutical Benefits Scheme (PBS) delisted the 2-mg tablet strength from public subsidy, reduced the pack size from 50 tablets to 10 tablets, and eliminated refills.^23^ Therefore, we used an interrupted time series analysis and cross-sectional study to determine whether these changes to the public subsidy of alprazolam were associated with in changes in (1) overall prescribing and dispensing, (2) patterns of prescribing and person-level use, or (3) poisoning calls to a poison information center (PIC) involving alprazolam.

## Methods

This study was approved by the New South Wales Population and Health Services Research Ethics Committee and the Sydney Children’s Hospital Network Human Research Ethics Committee. The requirement for informed consent was waived because the data were deidentified. Access to the PBS data was granted by the Australian Department of Human Services External Request Evaluation Committee. This study used the Strengthening the Reporting of Observational Studies in Epidemiology (STROBE) reporting guideline.

### Setting

All Australian citizens and residents are entitled to subsidized access to prescribed medications through the PBS. Some medications have restrictions on their prescribing through the PBS owing to their potential for harm or misuse; thus, alprazolam is an authority-required medication and can only be PBS subsidized if prior approval is obtained from the Department of Human Services. Medications can also be prescribed privately but are not PBS subsidized, and people must pay the entire cost of the medication out of pocket. Alprazolam is relatively inexpensive, with a pack of 10 tablets available for A$10 (US $6.76) or less.

### Intervention

In Australia, alprazolam is only PBS subsidized for panic disorder when other treatments have failed or are inappropriate.^24^ On February 1, 2017, multiple changes were made to the authority-required PBS listing of alprazolam. First, the 2-mg tablet strength was delisted from subsidy, but it could still be dispensed through private (ie, nonsubsidized) prescriptions. Second, the number of refills was reduced from 2 to 0, with no possibility of an increase; thus, people were required to visit a prescriber for each pack dispensed. Third, the pack size was reduced from 50 tablets to 10 tablets, but prescribers could request approval for larger amounts if deemed clinically necessary.^23^ Prescriptions written prior to February 1, 2017, could still be dispensed after this date if they were still valid.

### Data Sources

We used dispensing records from the PBS for a 10% random sample of Australian residents who had ever been dispensed alprazolam; this is a standard data set provided by the Australian Department of Human Services for analytical use and is selected based on the last digit of each individual’s randomly assigned unique identifier. This data set captures original and repeated dispensings for PBS-listed medications. Private dispensings are not captured. Prescribing approvals (ie, authorities) for all alprazolam PBS-subsidized prescriptions are recorded in a separate database by date of approval (ie, the date of prescribing), regardless of whether they were subsequently dispensed. To protect privacy, all dates for both data sets are randomly offset by −14 or +14 days; the direction of the offset is the same for all records for each individual. We used data from February 1, 2013, to December 31, 2018.

We also used data from telephone calls involving alprazolam exposure to a PIC from February 1, 2015, to October 31, 2018, to identify changes in poisonings associated with alprazolam. Australian PICs provide advice to health care professionals and members of the public 24 hours per day, 7 days per week. We used data from Australia’s largest PIC, New South Wales PIC, which takes approximately 50% of the nation’s 205 000 poisoning telephone calls per year. This includes calls from the states of New South Wales and Tasmania and the Australian Capital Territory, with after-hours calls taken from all of Australia 7 days per 2 weeks as part of the national roster.^25^

### Cohort of Alprazolam-Treated People

To identify changes in patterns of prescribing as well as person-level use of alprazolam during 1 year, we identified all people who had been dispensed alprazolam between August 1, 2017, and July 31, 2018, as the postintervention cohort. We excluded the first 6 months after the intervention to ensure that all dispensed alprazolam had been prescribed after the intervention date, as people could still refill prescriptions written prior to the intervention up to 6 months after the date of prescribing. As the comparison group, we identified all people dispensed alprazolam between November 1, 2015, and October 31, 2016, as the preintervention cohort. We excluded the 3 months leading up to the intervention because prescriber behavior may have changed in anticipation of the intervention, as we observed with the rescheduling of alprazolam in 2014.^26^ A new dispensing of alprazolam without a dispensing in the previous 730 days was considered initiation of a new treatment episode.

### Medications of Interest

Prior to February 1, 2017, alprazolam was available in the following tablet strengths: 0.25 mg, 0.5 mg, 1 mg, and 2 mg. After February 1, 2017, the 2-mg tablet was no longer subsidized. Alprazolam is relatively inexpensive, and it was still possible to obtain the 2-mg tablet strength alprazolam on the private market. During the study, among people receiving additional government benefits, such as those 65 years or older or with low incomes, the cost of alprazolam was lower if accessed via the PBS. However, for people who were not receiving government benefits, the out-of-pocket costs for a PBS-subsidized prescription or a private prescription were comparable.

To ensure that any observed changes were associated with the subsidy changes and not other interventions or nonspecific changes to dispensing and prescribing practices, we examined dispensing and prescribing for several control medications. As controls for changes in dispensing, we included dispensing of other subsidized benzodiazepines (ie, diazepam, oxazepam, nitrazepam, and temazepam). Benzodiazepines other than alprazolam generally do not require prior approval to prescribe; thus, as a control for prescribing approvals we included approvals for all medications (excluding alprazolam) that required an approval for the duration of the study.

Among the preintervention and postintervention cohorts, we also identified the dispensing of other medications for mental health disorders within the year using World Health Organization Anatomical Therapeutic Chemical classification codes,^27^ including other benzodiazepines (N05BA, N05CD), antidepressants (N06A), antipsychotics (N05A, excluding lithium), and gabapentinoids (pregabalin, gabapentin). We also examined dispensing of opioids (N02A), as their use in combination with benzodiazepines is generally not recommended and increases the risk of mortality in overdose.^13^

### Statistical Analysis

For each calendar month, we summed the number of dispensings and prescribing authority approvals for alprazolam and the control medications. As our data had no negative values, we log transformed our data to estimate relative (percentage) changes rather than absolute changes. We used an interrupted time series analysis to assess the change in each of these measures using a linear regression model with autoregressive errors, which includes previous (lagged) values of the outcome as predictors in the model to adjust for serial correlation of residuals. The number of dispensings and prescribing approvals for each medication was modeled separately, and the autoregressive order (ie, number of lagged values) for each model was chosen to maximize model fit (eg, Akaike Information Criterion) and to eliminate autocorrelation of residuals, as determined by the Durbin-Watson test and the autocorrelation function plot. To control for seasonality, we included monthly dummy variables. We considered both sudden, sustained changes (ie, level shift or step change), and changes in slope (ie, change in trend). We excluded the 6 months following the intervention, which we considered as a transitional period when people could still obtain refills for prescriptions written prior to February 1, 2017.

Among our preintervention and postintervention alprazolam-treated cohorts, we also examined changes in the prescribing patterns by calculating the proportion of all dispensings by number of tablets (≤10, 11-50, 51-100, >100) and total combined strength of all tablets (≤10 mg, 11-50 mg, 51-100 mg, >100 mg). We did this both overall and among new treatment episodes only. Owing to a relatively smaller cohort of new treatment episodes, we used the categories 10 or fewer, 11 to 50, and more than 50 for both tablets and total combined strength of all tablets. Categories were chosen to reflect the most common pack sizes. Because these proportions were relatively stable (no trend) through time (eFigures 1-4 in the Supplement), we compared changes using a before and after approach rather than an interrupted time series approach. We compared changes in prescribing patterns and characteristics of people receiving alprazolam treatment by calculating risk differences (RDs) with 95% CIs adjusted for correlation within individuals appearing in both the preintervention and postintervention cohorts.

Additionally, for each quarter, we calculated the number of telephone calls involving alprazolam to New South Wales PIC and the proportion of telephone calls involving the 2-mg tablet strength as a proxy for its use outside of the PBS-subsidized prescriptions. We analyzed these data using an interrupted time series analysis adjusted for autocorrelation only, as there was no significant seasonality in these data.

All analyses were performed with SAS statistical software version 9.3 (SAS Institute) and R version 3.5.1 (R Project for Statistical Computing). *P* values were 2-tailed, and statistical significance was set at .05. Data analyses were conducted from November 2018 to May 2019.

## Results

### Overall Dispensing and Prescribing Authority Approvals

**Figure 1.**
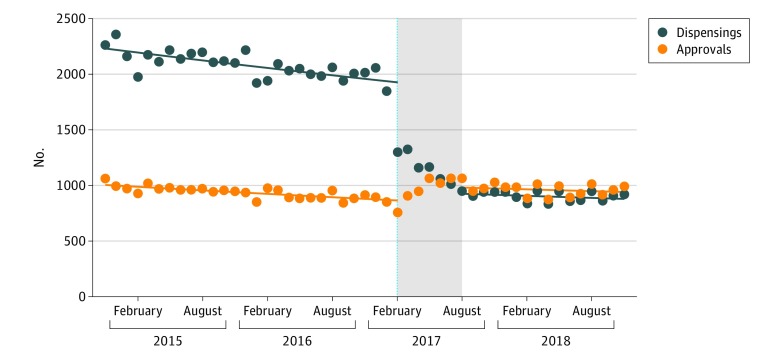
Dispensings and Prescribing Approvals for Alprazolam by Month The dashed line indicates the date of the subsidy changes (February 1, 2017); gray area, the 6 months after the intervention that were excluded from the model.

From January 1, 2015, to December 31, 2018, there were 71 481 alprazolam dispensings to 6772 people. After the intervention, the total number of alprazolam dispensings decreased by 51.2% (95% CI, 50.5%-51.9%) from August 1, 2017, to December 31, 2018, but there was a 17.5% (95% CI, 13.0%-22.2%) increase in prescribing approvals, as after the intervention, every pack dispensed required an approval (Table 1 and Figure 1). We observed only a small decrease in temazepam dispensing but no statistically significant changes in dispensing of other benzodiazepines or other prescribing approvals (eFigure 5, eFigure 6, eTable 1, and eTable 2 in the Supplement).

**Table 1.** Change in Monthly Dispensings and Prescribing Approvals After Change to Alprazolam Subsidy on February 1, 2017

Measure	Estimate, % (95% CI)	
	Dispensings	Prescribing Approvals
Monthly baseline trend	−0.5 (−0.6 to −0.5)	−0.6 (−0.7 to −0.4)
Level shift after subsidy change	−51.2 (−51.9 to −50.5)	17.5 (13.0 to 22.2)
Change in monthly trend	0.2 (0.1 to 0.3)	0.3 (0 to 0.6)

### Changes in Patterns of Prescribing

**Figure 2.**
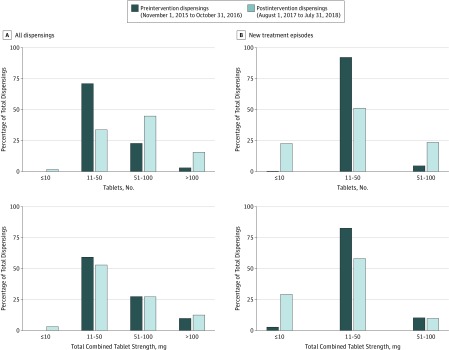
Dispensings by Number of Tablets and Total Combined Strength of all Tablets

In the preintervention cohort, there were 24 282 dispensings among 4212 people, of which 6874 dispensings (28.3%) were for the 2-mg tablet strength. In the postintervention cohort, there were 10 676 dispensings to 2419 people. In the preintervention cohort, 17 435 dispensings (71.8%) were for 11 to 50 tablets, followed by 5776 of 24 282 dispensings (23.8%) for 51 to 100 tablets (Figure 2A). Only 42 dispensings (0.2%) were for 10 tablets or fewer, and 1029 of 24 282 dispensings (4.2%) were for more than 100 tablets. After the intervention, the proportion of dispensings for 11 to 50 tablets decreased to 3706 (34.7%) (RD, −37.1% [95% CI, −39.4% to −34.7%]), and the proportion of dispensings for 51 to 100 tablets increased to 4888 of 10 676 dispensings (45.8%) (RD, 22.0% [95% CI, 19.4%-24.6%). The proportion of dispensings for 10 tablets or fewer, the new pack size, increased to 308 (2.9%) (RD, 2.7% [95% CI, 2.3%-3.2%), while the proportion of dispensings for more than 100 tablets increased to 1774 of 10 676 dispensings (16.6%) (RD, 12.4% [95% CI, 10.6%-14.2%]). Despite the delisting of the 2-mg tablet strength, there was little change in the total combined tablet strength per dispensing.

Among people receiving alprazolam treatment for the first time (ie, people with no dispensings within the previous 730 days) before the intervention, nearly all initial dispensings were for 11 to 50 tablets (1048 dispensings [93.0%]), reflecting the standard pack size of 50 tablets (Figure 2B). After the intervention, initiation with 10 tablets or fewer increased from 16 of 1127 people (1.4%) to 139 of 589 people (23.6%) (RD, 22.2% [95% CI, 18.7%-25.7%]), but initiation with more than 50 tablets also increased from 63 of 1127 people (5.6%) to 144 of 589 people (24.4%) (RD, 18.9% [95% CI, 15.1%-22.6%]).

### Changes in Person-Level Use

In the preintervention period, 4212 people were dispensed alprazolam, compared with 2419 people in the postintervention period, a 42.6% reduction. People who were dispensed alprazolam before the intervention received a median (interquartile range [IQR]) of 4 (1-9) dispensings, a median (IQR) total of 250 (50-600) tablets, and a median (IQR) total combined tablet strength of 188 (50-550) mg during 1 year; after the intervention, people received a median (IQR) of 3 (1-7) dispensings, a median (IQR) total of 200 (50-500) tablets, and a median (IQR) total combined tablet strength of 120 (30-360) mg.

Despite this reduction, 318 people (13.2%) received 10 or more dispensings, 583 people (24.1%) received more than 500 tablets, and 430 people (17.8%) received a total combined tablet strength of more than 500 mg during the year after the intervention (Table 2). Dispensings of other medications among people receiving alprazolam were proportionately similar before and after the intervention even after adjusting for age and sex, with high rates of people receiving dispensing of other benzodiazepines (RD, −0.6% [95% CI, −2.7% to 1.4%]), antidepressants (RD, 1.1% [95% CI, −0.7% to 3.0%]), antipsychotics (RD, 0.8% [95% CI, −0.7% to 2.6%]) and gabapentinoids (RD, 0.9% [95% CI, −0.5% to 2.3%]). Nearly half of the cohort were dispensed opioids, which was similar before and after the intervention (RD, 2.0% [95% CI, −0.2% to 4.1%]).

**Table 2.** Characteristics of People Who Were Dispensed Alprazolam During 1-Year Periods Before the Intervention and After the Intervention

Characteristic	No. (%)		Age- and Sex-Adjusted Risk Difference, % (95% CI)
	Preintervention Cohort^a^ (n = 4212)	Postintervention Cohort^b^ (n = 2419)	
Age, median (IQR), y	55 (41-68)	58 (44-71)	NA
Sex			
Men	1586 (37.7)	858 (35.5)	−1.6 (−3.3 to 0.1)
Women	2626 (62.4)	1561 (64.5)	1.6 (−0.1 to 3.3)
Alprazolam-naive^c^			
Yes	1124 (26.7)	587 (24.3)	−0.1 (−3.0 to 1.0)
No	3088 (73.3)	1832 (75.7)	0.1 (−1.0 to 3.0)
Dispensings, No.			
1	1122 (26.6)	791 (32.7)	7.2 (5.1 to 9.3)
2-4	1015 (24.1)	670 (27.7)	3.5 (1.4 to 5.5)
5-9	1136 (27.0)	640 (26.5)	−1.2 (−3.3 to 0.8)
≥10	939 (22.2)	318 (13.2)	−9.4 (−11.0 to −7.9)
Maximum tablet strength dispensed, mg			
0.25	675 (16.0)	339 (14.0)	−2.3 (−3.7 to −0.8)
0.5	1215 (28.9)	847 (35.0)	5.6 (3.8 to 7.4)
1	1355 (32.2)	1233 (51.0)	18.8 (16.8 to 20.8)
2	967 (23.0)	0	NA
Total tablets dispensed, No.			
≤50	1065 (25.3)	620 (25.6)	1.9 (−0.3 to 3.6)
51-200	974 (23.1)	654 (27.0)	3.9 (1.8 to 5.9)
201-500	1016 (24.1)	562 (23.2)	−1.5 (−3.4 to 0.4)
>500	1157 (27.4)	583 (24.1)	−3.7 (−5.4 to −2.0)
Total combined tablet strength, mg			
≤50	1275 (30.3)	850 (35.1)	5.4 (3.3 to 7.5)
51-200	1063 (25.2)	668 (27.6)	1.9 (−0.1 to 3.9)
201-500	774 (18.4)	471 (19.5)	0.8 (−0.9 to 2.5)
>500	1100 (26.1)	430 (17.8)	−7.9 (−9.5 to −6.4)
Other medications dispensed			
Other benzodiazepines	1548 (36.8)	846 (35.0)	−0.6 (−2.7 to 1.4)
Antipsychotics	781 (18.5)	458 (18.9)	0.8 (−0.7 to 2.6)
Antidepressants	2860 (67.9)	1670 (69.0)	1.1 (−0.7 to 3.0)
Gabapentinoids	521 (12.4)	328 (13.6)	0.9 (−0.5 to 2.3)
Opioids	1769 (42.0)	1065 (44.0)	2.0 (−0.2 to 4.1)

Abbreviations: IQR, interquartile range, NA, not applicable.

^a^ Includes 10% sample of people who were dispensed alprazolam subsidized by Pharmaceutical Benefits Scheme from November 1, 2015 to October 31, 2016.

^b^ Includes 10% sample of people who were dispensed alprazolam subsidized by Pharmaceutical Benefits Scheme from August 1, 2017, to July 31, 2018.

^c^ Defined as not having received an alprazolam prescription in the past 730 days.

### Changes in Calls to PIC

From February 1, 2015, to October 31, 2018, there were 1096 calls to New South Wales PIC involving alprazolam; 938 calls (85.6%) were for intentional misuse (eg, recreational use, deliberate self-poisoning), 130 calls (11.9%) were for unintentional misuse (eg, adverse reactions, therapeutic errors), and 28 calls (39.1%) could not be classified. Of 606 calls (55.3%) in which the total dose ingested was recorded, the median (IQR) dose before the intervention was 9 (3-25) mg; after the intervention, the median (IQR) dose was 10 (3-36) mg.

**Figure 3.**
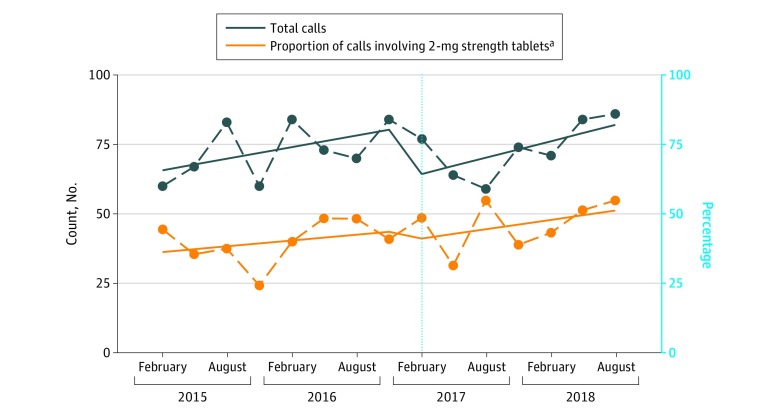
Calls to New South Wales Poisons Information Centre Involving Alprazolam by Quarter Total calls are measured as count; proportion of calls involving 2-mg tablets is shown as percentage. The dashed line indicates the date of the subsidy changes on February 1, 2017. ^a^Proportion of calls involving the 2-mg strength tablets was estimated among 505 calls (46.1%) in which this information was recorded.

There was a median (IQR) of 72 (64-84) calls per quarter before the intervention and 74 (64-84) calls per quarter after the intervention. The number of calls was stable before the intervention (baseline trend, increase of 2 calls per quarter [95% CI, 0 to 4 calls]) (Figure 3). We observed a slight decrease in calls after the intervention (level shift, −20 calls [95% CI, −35 to −3 calls]) but no change in trend (increase of 1 call [95% CI, −3 to 4 calls]). There were 505 calls (46.1%) with the tablet strength recorded. Of these, 215 calls (42.6%) involved the 2-mg tablet strength. This proportion was stable over time (baseline trend, increase of 1% [95% CI, −2% to 4%]). There was no level shift (−4% [95% CI, −22% to 13%]) or change in trend (increase of 1% [95% CI, −3% to 5%]).

## Discussion

Our study found a substantial 51.2% decrease in overall PBS dispensing of alprazolam after the introduction of the new prescribing restrictions; this was accompanied by an 17.5% increase in the number of approvals for new prescriptions. While we did observe a reduction in PBS-subsidized alprazolam use, which was the main target of the intervention, we estimate that after the intervention, there were approximately 2000 additional prescriptions per month at the population level, each requiring a visit to a prescriber plus an authority approval. Thus, it is unclear whether the observed reduction in dispensing will result in cost savings for the government given the increase in approvals to prescribe, and its impact on health outcomes is uncertain.

Notwithstanding the reduction in dispensings, among individuals who were still being dispensed alprazolam after the intervention, prescribers appeared to compensate for the elimination of 2-mg tablets and refills by prescribing increased quantities, despite the introduction of a new smaller pack size. Reducing the pack size, while well intended, was not sufficient to reduce quantities dispensed. Since all prescribers had to request approval to prescribe alprazolam, requesting approval for increased amounts required little extra effort, as it could be done at the same time. Thus, a substantial proportion of people were still receiving amounts well above the recommended dosages and durations of therapy.^28^ Even among people initiating new treatment courses, we observed an increase in dispensing of pack sizes of more than 50 tablets, putting people at a greater risk of transition to long-term use and dependence.

Similar approaches have been used to restrict access to other commonly misused medications, such as opioids and quetiapine, with equally mixed results. In the United States, when prescription refills were no longer available for hydrocodone after its rescheduling, the amounts dispensed at the initiation of postsurgery treatment increased.^29^ When refills for low-dose quetiapine were eliminated in Australia to prevent prescribing outside its approved indications, subsidized dispensing decreased overall, but there was little impact on inappropriate use.^5^ Despite the reduction in subsidized alprazolam use, we cannot be certain to what extent prescribing decreased overall, as we did not have data on nonsubsidized (ie, private) prescriptions. Given alprazolam’s low cost, a shift to the private market would not be surprising, as there is little price difference between private or PBS-subsidized prescriptions for many people. Likewise, in Ontario, Canada, after high-strength opioids were delisted from the public formulary, 1 in 3 people accessed them via private insurance or by paying for the medication out of pocket.^6^

A 2013 study by Nielsen et al^30^ suggested that most people who misuse benzodiazepines were prescribed them by a health care practitioner for the treatment of real symptoms, making prescribers an appropriate target for intervention. However, many prescribers are skeptical about certain policy interventions, such as requirements for authorities, to promote prescribing according to best-practice recommendations and safe use of medications.^31^ Moreover, discontinuing long-term benzodiazepine use can be difficult^32,33^; thus, increasing restrictions on subsidized benzodiazepine prescribing on its own may have minimal effect on problematic long-term use, particularly in the context of a thriving private market. Imprecise interventions, such as withdrawing reimbursement, often have unintended consequences and are rarely sufficient on their own to promote safer use of medications.^8^

### Limitations

Our study had some limitations. First, we had no information about indication for treatment or other clinical information for people who were dispensed alprazolam; thus, we could not assess appropriateness of prescribing. We also did not have information about the dispensing of alprazolam on the private market; in 2015, approximately one-quarter of alprazolam was prescribed privately,^34^ and this has likely increased in recent years. While some people may have switched to smaller PBS-listed tablet strengths after the intervention, there was no change in the proportion of calls to the PIC involving the 2-mg tablet strength. While these results suggest that the 2-mg tablet strength is still available in the community, calls to a PIC are a crude measure, only capture a subset of poisonings, and should be interpreted with caution. Further work will be needed to determine the true effect of the intervention on health outcomes.

## Conclusions

While this study found that the intervention was associated with a decrease in dispensing of PBS-subsidized alprazolam, there was an increase in prescribing approvals, potentially placing an increased burden on the health care system without clear benefits in reducing inappropriate use. Most people dispensed alprazolam were still receiving treatment outside the restrictions and best-practice recommendations, particularly with increased pack sizes to work around the reductions in refills per prescription. Further, the PIC data suggest that the intervention was associated with minimal changes to dispensing of the 2-mg tablet strength, as prescribing appears to have shifted to the private market.

## Notes

Supplement. **eFigure 1.** Proportion of All Dispensings by Tablets Dispensed by Month **eFigure 2.** Proportion of All Dispensings by Total Combined Strength of All Tablets Dispensed by Month **eFigure 3.** Proportion of All Dispensings by Tablets Dispensed to People Initiating a New Treatment Episode By Quarter **eFigure 4.** Proportion of All Dispensings by Total Combined Strength of all Tablets Dispensed to People Initiating a New Treatment Episode By Quarter **eFigure 5.** Dispensing of Benzodiazepines by Month **eTable 1.** Change in Monthly Dispensings of Benzodiazepines After Changes to Alprazolam Subsidy Estimated Using Autoregressive Models **eFigure 6.** All Prescribing Approvals Excluding Alprazolam by Month **eTable 2.** Change in Monthly Total Prescribing Approvals Excluding Alprazolam After Changes To Alprazolam Subsidy Estimated Using Autoregressive Models
