# Supplementary material for: Comparison of Prescribing Patterns Before and After Implementation of a National Policy to Reduce Inappropriate Alprazolam Prescribing in Australia
Source: JAMA Netw Open. 2019 Sep 18;2(9):e1911590. doi: 10.1001/jamanetworkopen.2019.11590 (PMC6751760; doi:10.1001/jamanetworkopen.2019.11590)
Supplement: Supplement. — eFigure 1. Proportion of All Dispensings by Tablets Dispensed by Month eFigure 2. Proportion of All Dispensings by Total Combined Strength of All Tablets Dispensed by Month eFigure 3. Proportion of All Dispensings by Tablets Dispensed to People Initiating a New Treatment Episode By Quarter eFigure 4. Proportion of All Dispensings by Total Combined Strength of all Tablets Dispensed to People Initiating a New Treatment Episode By Quarter eFigure 5. Dispensing of Benzodiazepines by Month eTable 1. Change in Monthly Dispensings of Benzodiazepines After Changes to Alprazolam Subsidy Estimated Using Autoregressive Models eFigure 6. All Prescribing Approvals Excluding Alprazolam by Month eTable 2. Change in Monthly Total Prescribing Approvals Excluding Alprazolam After Changes To Alprazolam Subsidy Estimated Using Autoregressive Models [file jamanetwopen-2-e1911590-s001.pdf]

Supplementary Online Content

Schaffer AL, Buckley NA, Cairns R, Pearson S. Comparison of prescribing patterns before and after implementation of a national policy to reduce inappropriate alprazolam prescribing in Australia. *JAMA Netw Open*. 2019;2(9):e1911590 doi:10.1001/jamanetworkopen.2019.11590

eFigure 1. Proportion of All Dispensings by Tablets Dispensed by Month

eFigure 2. Proportion of All Dispensings by Total Combined Strength of All Tablets Dispensed by Month

eFigure 3. Proportion of All Dispensings by Tablets Dispensed to People Initiating a New Treatment Episode By Quarter

eFigure 4. Proportion of All Dispensings by Total Combined Strength of All Tablets Dispensed to People Initiating a New Treatment Episode By Quarter

eFigure 5. Dispensing of Benzodiazepines by Month

eTable 1. Change in Monthly Dispensings of Benzodiazepines After Changes to Alprazolam Subsidy Estimated Using Autoregressive Models

eFigure 6. All Prescribing Approvals Excluding Alprazolam by Month

eTable 2. Change in Monthly Total Prescribing Approvals Excluding Alprazolam After Changes To Alprazolam Subsidy Estimated Using Autoregressive Models

This supplementary material has been provided by the authors to give readers additional information about their work.

© 2019 Schaffer AL et al. *JAMA Network Open.*

eFigure 1. Proportion of All Dispensings by Tablets Dispensed by Month

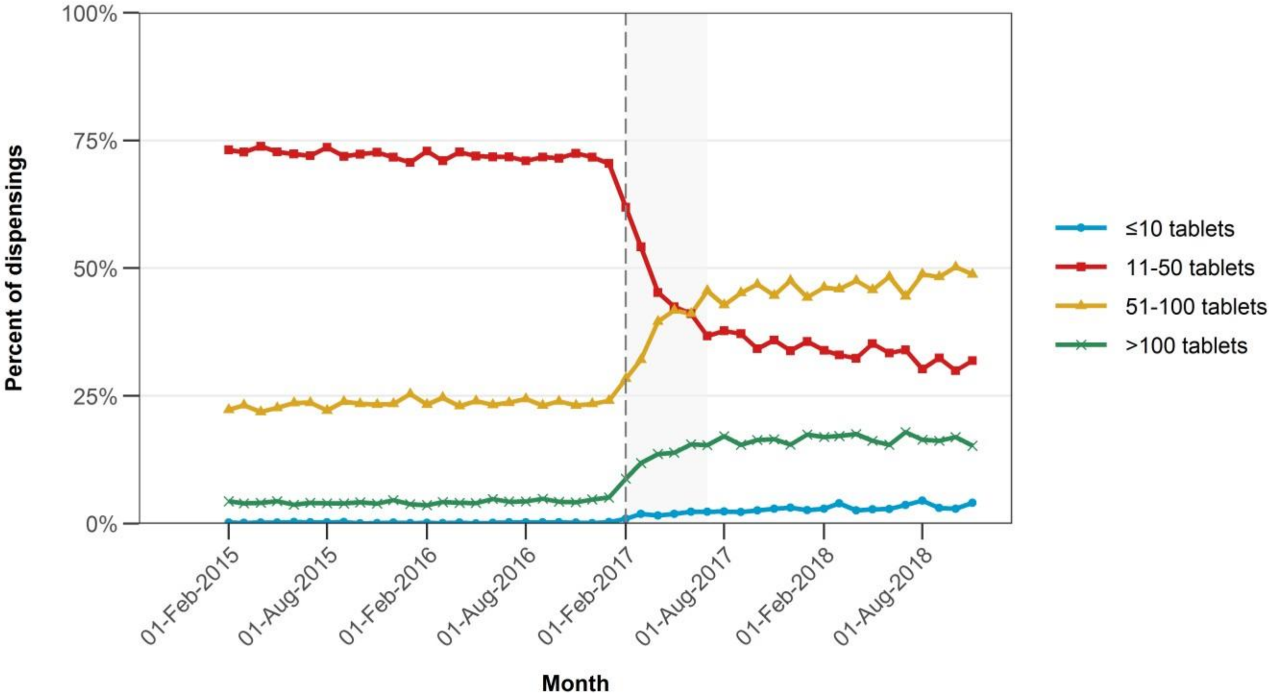

© 2019 Schaffer AL et al. *JAMA Network Open.*

eFigure 2. Proportion of All Dispensings by Total Combined Strength of All Tablets Dispensed by Month

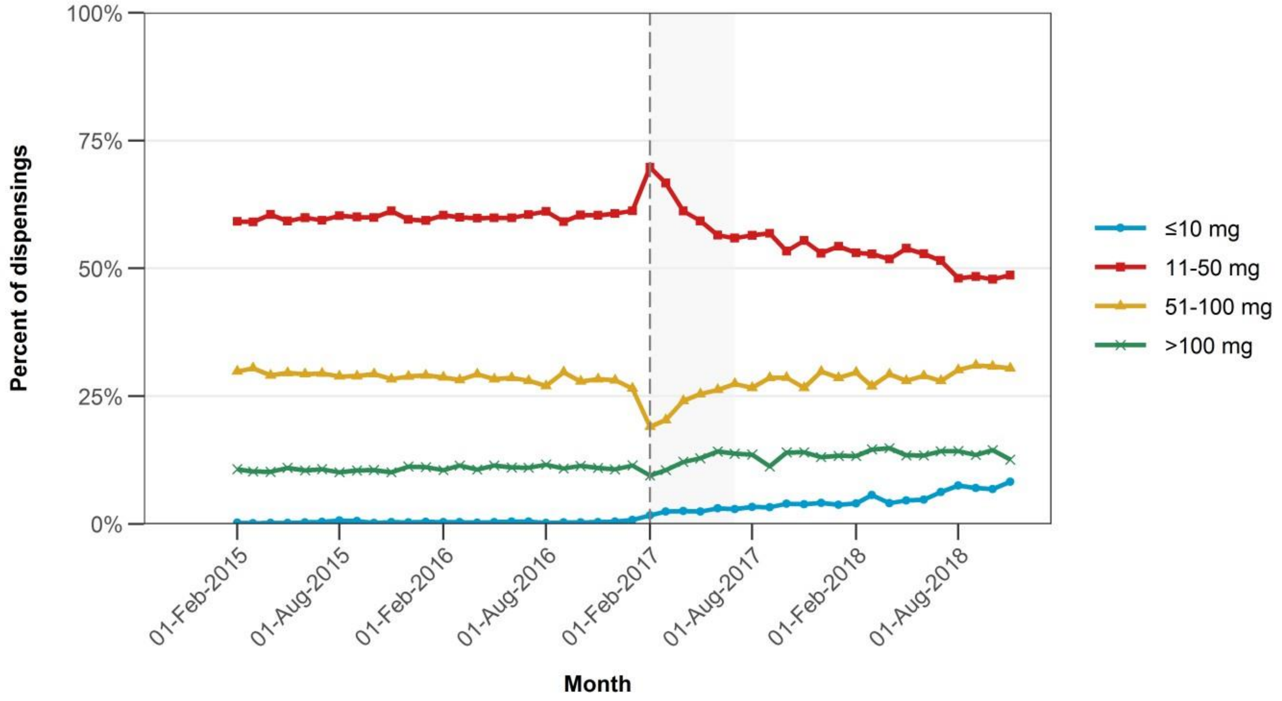

© 2019 Schaffer AL et al. *JAMA Network Open.*

eFigure 3. Proportion of All Dispensings by Tablets Dispensed to People Initiating a New Treatment Episode By Quarter

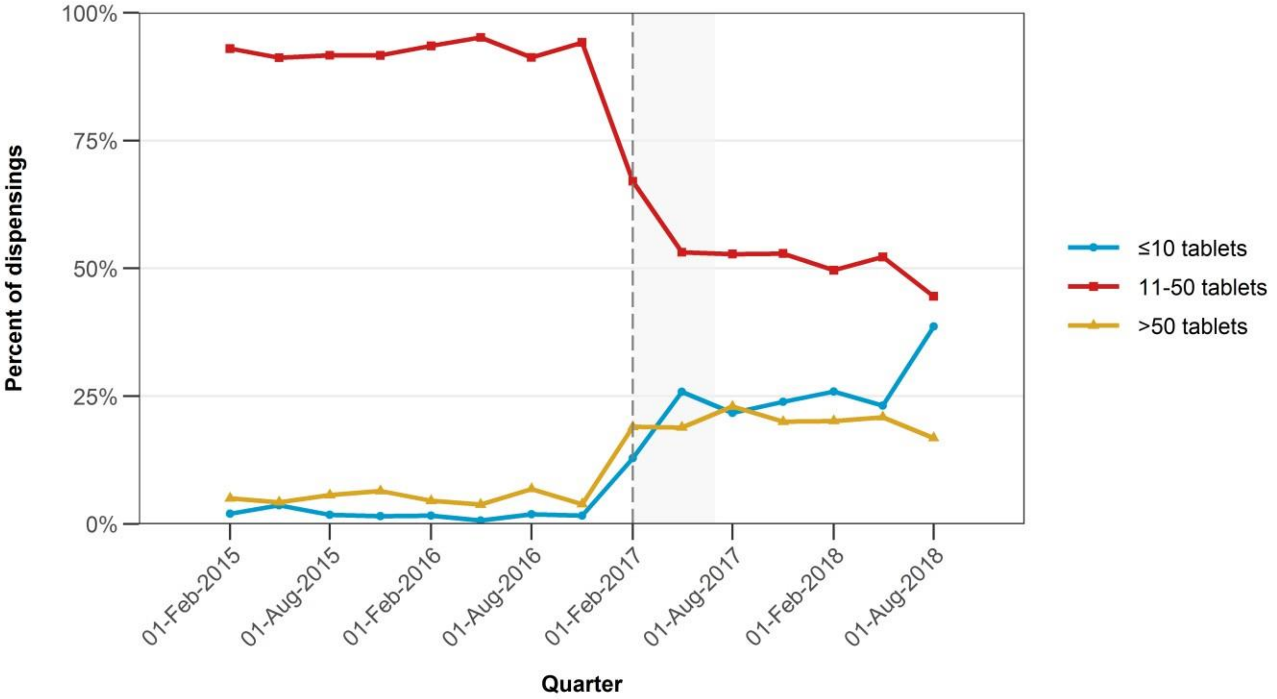

© 2019 Schaffer AL et al. *JAMA Network Open.*

eFigure 4. Proportion of All Dispensings by Total Combined Strength of All Tablets Dispensed to People Initiating a New Treatment Episode By Quarter

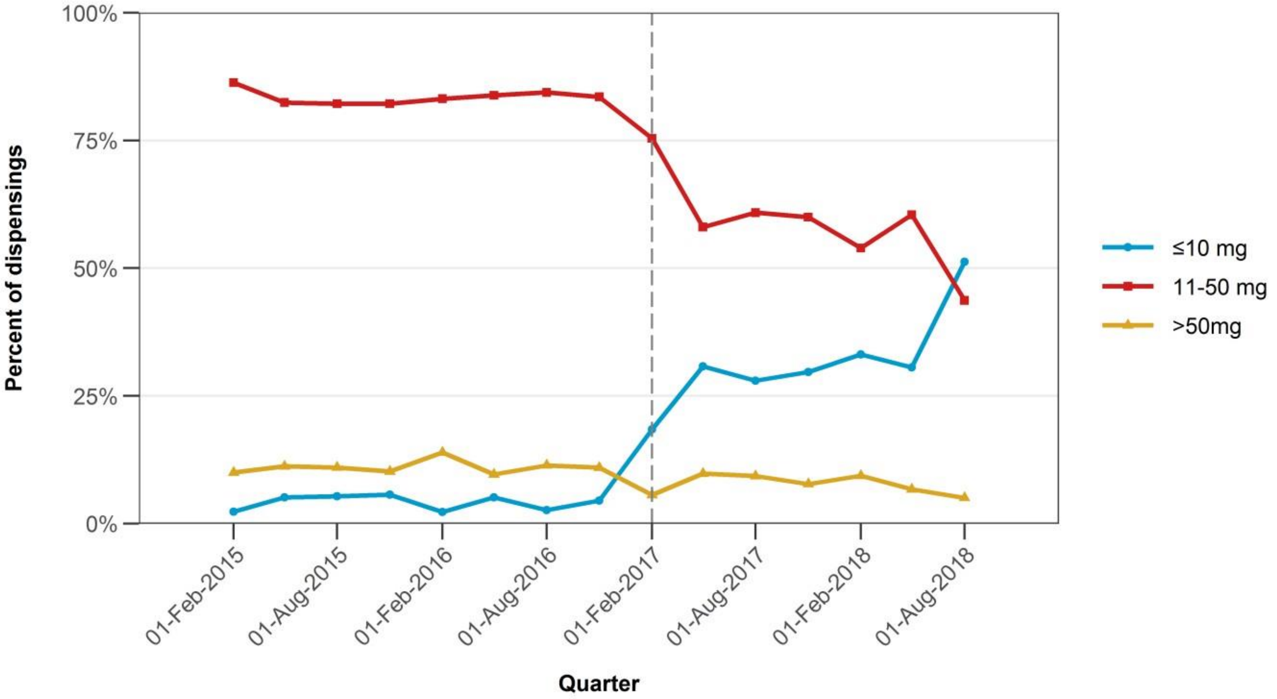

© 2019 Schaffer AL et al. *JAMA Network Open.*

eFigure 5. Dispensing of Benzodiazepines by Month

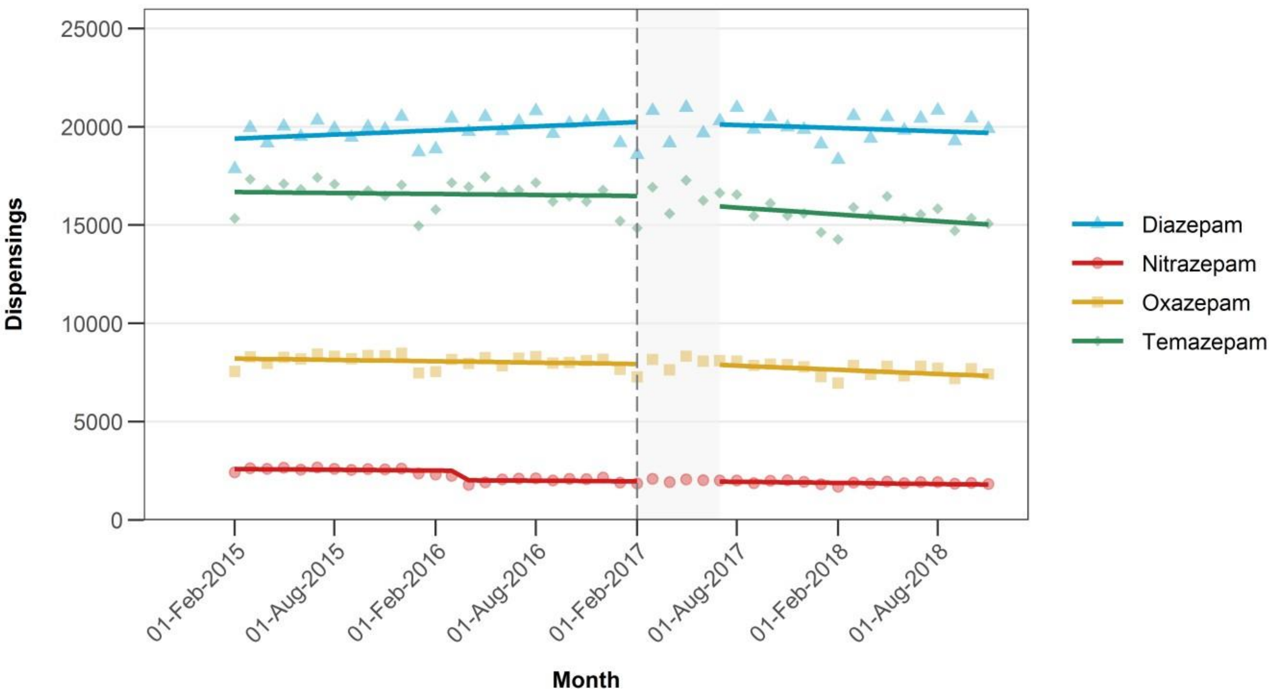

© 2019 Schaffer AL et al. *JAMA Network Open.*

eTable 1. Change in Monthly Dispensings of Benzodiazepines After Changes to Alprazolam Subsidy Estimated Using Autoregressive Models

	Diazepam		Nitrazepam		Oxazepam		Temazepam	
	**Estimate**	**95% CI**	**Estimate**	**95% CI**	**Estimate**	**95% CI**	**Estimate**	**95% CI**
**Monthly baseline trend**	0.2%	0.1%, 0.3%	-0.3%	-0.8%, 0.3%	-0.1%	-0.2, -0.1%	-0.1%	-0.1%, 0.0%
**Level shift after subsidy change**	-1.4%	-3.5%, 0.6%	0.6%	-7.4%, 9.3%	0.1%	-1.5%, 1.6%	-3.0%	-4.5%, -1.5%
**Change in monthly trend**	-0.3%	-0.5%, -0.2%	-0.3%	-1.1%, 0.6%	-0.3%	-0.4%, -0.2%	-0.3%	-0.4%, -0.2%

© 2019 Schaffer AL et al. *JAMA Network Open.*

eFigure 6. All Prescribing Approvals Excluding Alprazolam by Month

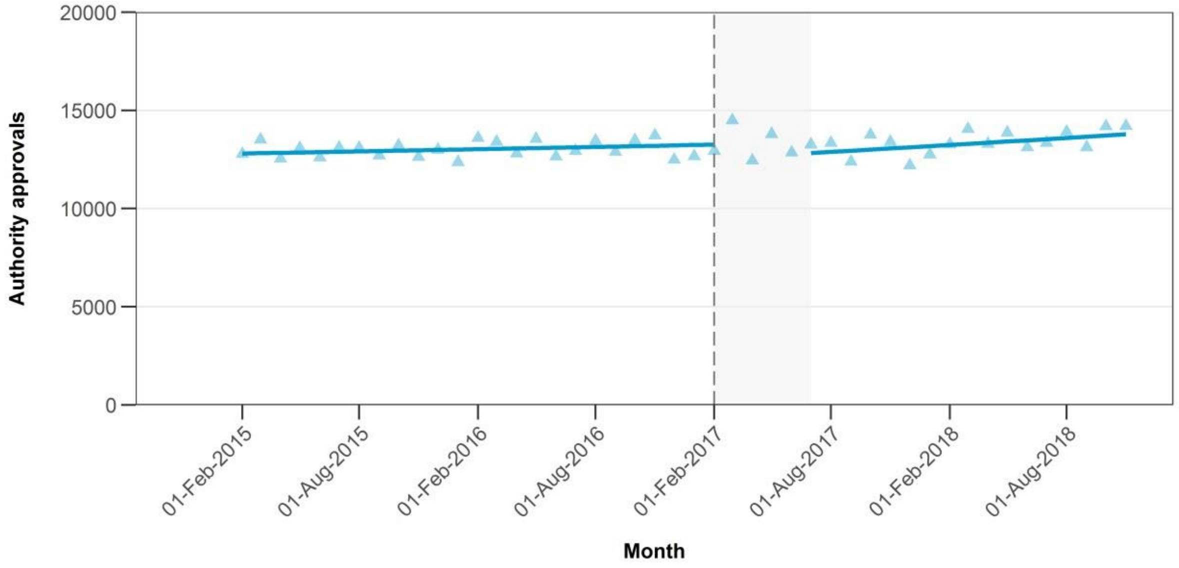

© 2019 Schaffer AL et al. *JAMA Network Open.*

eTable 2. Change in Monthly Total Prescribing Approvals Excluding Alprazolam After Changes To Alprazolam Subsidy Estimated Using Autoregressive Models

	Prescribing approvals, excluding alprazolam	
	**Estimate**	**95% CI**
**Monthly baseline trend**	0.2%	0.1%, 0.3%
**Level shift after subsidy change**	-1.4%	-3.5%, 0.6%
**Change in monthly trend**	-0.3%	-0.5%, -0.2%

© 2019 Schaffer AL et al. *JAMA Network Open.*
